# Facilitators and barriers to early-stage dementia care: a qualitative study on the perspectives of people with dementia, informal caregivers, and healthcare professionals

**DOI:** 10.3389/fpubh.2026.1867784

**Published:** 2026-07-06

**Authors:** Sanne C. E. Balvert, Romano D. de Vries, Rose Marie Dröes, Leonie N. C. Visser, Maarten V. Milders

**Affiliations:** 1 Department of Clinical Neuropsychology, Faculty of Behavioural and Movement Sciences, Vrije Universiteit, Amsterdam, Netherlands; 2 Alzheimer Center Amsterdam, Neurology, Vrije Universiteit Amsterdam, Amsterdam UMC, Amsterdam, Netherlands; 3 Department of Psychiatry, VU University Medical Center, Amsterdam UMC, Amsterdam, Netherlands; 4 Amsterdam Publich Health Research Institute, Amsterdam UMC Location VUmc, Amsterdam, Netherlands; 5 Department of Medical Psychology, Amsterdam UMC, Amsterdam, Netherlands; 6 Department of Bioethics and Health Humanities, Julius Center for Health Sciences and Primary Care, University Medical Center Utrecht, Utrecht University, Utrecht, Netherlands

**Keywords:** barriers, early-stage dementia care, facilitators, healthcare professionals, Informal caregivers, qualitative content analysis

## Abstract

**Background:**

While timely support can benefit people with dementia and their informal caregivers, the period between recognizing first symptoms and receiving a formal diagnosis is often prolonged. Barriers like stigma, denial, and symptom misinterpretation hinder care acceptance, leaving many without adequate support. This study aimed to (i) explore the needs of community-dwelling people with dementia and informal caregivers; (ii) gain insight into facilitators and barriers for support; and (iii) identify strategies used by healthcare professionals to improve early-stage care acceptance, and whether geographical context influenced this.

**Methods:**

This qualitative study used a cross-sectional design. We recruited 35 dyads of persons with dementia (mean age 77.7 ± 7 years; 51% female) and informal caregivers (70.0 ± 14 years; 60% female) through daycare centers and memory clinics in the Netherlands. Healthcare professionals (*N* = 47; 87% female; 38% dementia case manager) were contacted via regional dementia networks. Open-ended questionnaires assessed dyads' experiences and needs, while healthcare professionals participated in semi-structured interviews. Audio-recorded interviews were transcribed. Thematic content analysis was used on all data by two independent coders.

**Results:**

Retrospectively, dyads indicated a strong need for timely knowledge, practical resources, and peer support in the early stage. Dementia case managers were considered crucial for accessing support. Effective strategies for professionals to improve early-stage care acceptance included: community engagement, raising societal awareness through education, a personal approach based on patience and trust, and better collaboration with other organizations to maintain short lines of communication. Barriers included reluctance to seek help, stigma, limited awareness, misalignment of services and needs, bureaucratic complexity, and difficulty navigating the healthcare system. Professionals stressed the need for tailored outreach strategies to both individual needs and local context, given perceived differences between city and rural regions in the effectiveness of strategies and dyads' needs.

**Conclusion:**

Our findings suggest that trust-based, relational approaches, characterized by patience, personal contact, and low-threshold access, are central to facilitating early-stage care acceptance. Early-stage support should be made more visible in communities and tailored to individual needs and local context. Reducing stigma and bureaucratic complexity, while strengthening local facilitators, may improve access to timely and meaningful support.

## 1 Introduction

As the global population ages, the prevalence of dementia is rising at an alarming rate. The number of people with dementia is projected to nearly triple to 152 million by 2050 (1). An early diagnosis of dementia enables timely access to information, support, services, and appropriate care. It provides clarity for people with dementia and their families, and more importantly, allows people with dementia to participate in informed decision-making while they still can (2, 3). People with dementia often express a desire to preserve their autonomy and remain independent and in control for as long as possible (4, 5). Receiving a timely diagnosis gives people with dementia and their families the opportunity to plan for the future, seek legal and financial advice, and to receive timely support (2). An earlier diagnosis can also enable the person with dementia to adopt healthier lifestyle changes, which may help optimize overall health and wellbeing (6). Timely use of community-services and support can also help delay the institutionalization of the person with dementia (4, 7). Early recognition of dementia thus offers several advantages that can improve the quality of life of people with dementia, as well as of their informal caregivers and relatives (2). Previous research has shown that support provided at an early stage was more effective in improving wellbeing of the caregiver than support offered at a later stage of dementia (8). Moreover, the timing of when people with dementia and their informal caregivers accept professional support and care is crucial, as it can influence the trajectory of the caregiving situation (4). Early-stage intervention may better equip all parties involved to cope with dementia progression and adapt to changes in family dynamics at home, as people with dementia at this stage can still fully contribute and express their needs and preferences (2, 9). Healthcare professionals recognize the importance of timely support in dementia care and employ various strategies to facilitate the acceptance of care (4). Nevertheless, these efforts are not always successful, and professionals often express concerns that support is provided too late to be truly effective. Geographical context may further shape access to early-stage dementia care, as regional differences in healthcare infrastructure and service availability can influence how easily people with dementia and their informal caregivers find and use available support (10–12). Rural populations in particular are often at a disadvantage, with fewer services available to them and greater distances to travel (11).

There is often a prolonged period between recognizing the first symptoms and receiving a formal diagnosis, which can range from several months to several years, also known as the diagnostic delay (13). Several factors contribute to this delay in diagnosis, including individuals being reluctant to seek help due to barriers such as stigma, denial or fear (4, 5, 14), insufficient knowledge about dementia, and a lack of awareness of available services (4, 14), including lack of awareness among general practitioners (15, 16). There is still a lack of knowledge about how to overcome these barriers effectively. Early symptoms can also frequently be mistaken for normal aging, which can lead to misinterpretation by general practitioners, resulting in a high rate of underdiagnosis in primary care (2, 17). Research has shown that while people with dementia frequently report needing help in coming to terms with their situation or finding strategies to cope with their difficulties, they often do not have a clear idea of how these needs should be met, which may further complicate timely help-seeking and support (18). Consequently, the pre-diagnostic and help-seeking period in dementia can be a long and challenging experience. Even after receiving a diagnosis, informal caregivers may postpone or avoid the use of formal care, because they do not feel the support is necessary, even when experiencing significant burden (4, 19). Additionally, informal caregivers may refrain from seeking help because they believe that caring for a family member with dementia is their personal or family responsibility (14). The non-utilization of services is further influenced by the reluctance of the person with dementia to accept care, fear of losing autonomy, and lack of awareness of the existing services available (4, 5, 20, 21). Furthermore, people with dementia and their caregivers residing in more disadvantaged regions often have less awareness of, and fewer options for, available support (10). As a result, by the time a person with dementia or their informal caregiver seeks or receives formal care, it is often too late for optimal support (4). By that time, the difficulties at home may already be so severe that community-based care has limited effectiveness, and admission to residential care may soon follow (7).

To improve the timing and acceptance of care, it is essential to better understand the needs of people in the early stages of dementia and the barriers that prevent them from seeking or accepting help. In this study, the term early-stage care acceptance refers to a continuum of engagement with care, from the recognition of first symptoms and the willingness to seek a formal diagnosis, to the acceptance and use of professional support following that diagnosis. Developing strategies to facilitate earlier diagnosis and intervention is crucial for improving symptom management, decreasing caregiver burden, reducing costs, and delaying institutional care. However, active involvement of the person with dementia in designing early-stage interventions is limited, despite its potential to enhance acceptance and uptake (22). For this reason, we explored the perspectives of all stakeholders involved: people with dementia, their informal caregivers, and healthcare professionals. The goals of this study were to explore the experiences of community-dwelling people with dementia and their informal caregivers in the early stages of the disease to examine their needs regarding early-stage support, and what hindered or enabled their access to care. We also examined how healthcare professionals try to reach people with dementia and their caregivers, by collecting information on both successful and unsuccessful strategies employed by healthcare and welfare organizations to facilitate timely use of available services. To this end, this study aimed not only to identify what is needed and what works, but to understand how needs, facilitators, and barriers interacted across stakeholder groups and geographical contexts, and what these dynamics meant for the timing and acceptance of early-stage dementia care. In doing so, this study sought to provide insights that could contribute to improving early-stage dementia care and ultimately enhance the quality of life for those affected and their informal caregivers.

## 2 Methods

### 2.1 Design

This qualitative study was part of a larger research project called “Eerder Erbij” (ZonMw grant number: 852002106). The current study used a cross-sectional design and collected information on: (i) the needs of community-dwelling people with dementia and their informal caregivers in the early stages of dementia; (ii) the facilitators and barriers for support; and (iii) strategies used by healthcare professionals to improve the acceptance of early-stage care. Open-ended questionnaires were used to assess the perspectives of the person with dementia and their informal caregiver, hereinafter referred to as dyad, and semi-structured interviews were held to examine the perspectives of healthcare professionals. Data collection took place during the COVID-19 pandemic, during which physical contact with people with dementia was avoided where possible. As many people with dementia were not yet sufficiently familiar with digital tools to participate in online interviews, paper questionnaires were used.

### 2.2 Ethical approval

The “Eerder Erbij” study was conducted according to the principles of Helsinki, and all participants gave informed consent. No interview was conducted before obtaining written informed consent. The study was declared by the Medical Ethics Committee of the Amsterdam University Medical Centre (METC-2021.0295) as research not subjected to the Medical Research Involving Human Subjects Act.

### 2.3 Participants and procedure

All participants were recruited between July 2021 and June 2022, which coincided with the global pandemic caused by COVID-19. Due to the restrictions on physical and social contact, data were collected either through postal questionnaires or through semi-structured interviews conducted online.

#### 2.3.1 People with dementia and informal caregivers

The people with dementia and caregivers were recruited through the older populations care organizations, such as dementia daycare centers and memory clinics. They were approached by healthcare professionals, who informed potential participants of the study and provided more information on what participation entailed. When interested, dyads received written information explaining the study and an informed consent form. Upon receipt of a signed informed consent form, a questionnaire was sent. Due to COVID-19 restrictions at the time of data collection, participants were asked to complete the questionnaires at home, but they could contact the research team by phone if they had any questions or required assistance. To map out the wishes and needs of the people with dementia and their caregivers as broadly as possible, the aim was to recruit around 36 dyads.

To be eligible for the study, the person with dementia had to live at home, and had to have a dementia diagnosis or experience severe cognitive impairment suggesting dementia and had to be able to give informed consent. The informal caregiver could be the partner or a child of the person with dementia. If the caregiver did not live with the person with dementia, they had to care for the person with dementia at least three times a week without receiving payment. Participants were recruited as dyads and were asked to fill out the questionnaire together.

#### 2.3.2 Healthcare professionals

Healthcare professionals were recruited through regional dementia networks and a network of daycare- and healthcare organizations that had been involved in a previous study (23) and through snowball sampling. After giving informed consent, each professional was invited to participate in an interview. The interviews were conducted online by two members of the research team (SB and HdV; researcher and research assistant, both female). All interviews were audio-recorded and lasted approximately 1 h on average. If healthcare professionals were unable to participate in an interview due to time- or other constraints, they were asked to complete a survey containing the same questions as the interview. The aim was to approach 30 healthcare professionals from different occupational backgrounds in order to capture a broad range of perspectives regarding support in early-stage dementia.

Inclusion criteria for the healthcare professionals were that they had to work for an older adults care organization, e.g., dementia daycare center, district nurse or domestic care, where they had experience in working with community-dwelling people with dementia and informal caregivers. Healthcare professionals whose only experience with people with dementia was in residential care were not included in the study.

### 2.4 Materials

#### 2.4.1 Surveys for people with dementia and informal caregivers

Dyads completed a structured survey including sociodemographic characteristics, such as age, gender, educational level, and relationship to each other. The person with dementia provided additional information regarding their diagnosis, while the informal caregiver was asked about other responsibilities in addition to caregiving (e.g., work or family obligations). The survey further addressed needs for support when symptoms first emerged and at the time of diagnosis, including the type of help that would have been useful in the early stages, preferred timing of support throughout the course of the disease, and the type of services actually used at that time. Participants were also asked how healthcare professionals could have assisted them in the early stages, and whether they encountered difficulties in finding help during that time. All survey questions are available in Supplementary Appendix A.

#### 2.4.2 Semi-structured interviews with healthcare professionals

Healthcare professionals were interviewed using a semi-structured guide. Initial questions addressed socio-demographic and work-related characteristics, including the region where they worked, their professional role, and years of experience in older adults care. Subsequently, questions focused on strategies to reach people with dementia and their informal caregivers at an early stage, collaborations with other organizations to support these efforts, and ways to identify care needs upon initial contact. Professionals were also asked to reflect on barriers and facilitators to early engagement, as well as to evaluate which strategies were considered successful or unsuccessful in promoting early-stage care acceptance. See Supplementary Appendix B for all interview questions.

### 2.5 Data analysis

The audio-recorded interviews were transcribed by research assistants, who were not involved in the data collection. All transcripts were subsequently checked for accuracy by SB. To support the analysis, the qualitative analysis software MAXQDA (VERBI Software, 2024) was used to assist in the coding process and identification of themes. An inductive, thematic content analysis was conducted, following the approach of Vears and Gillam (24), in which codes and categories are derived directly from the data. This study was designed from a constructivist epistemological perspective, recognizing that participants' experiences are shaped by their personal and contextual circumstances. To reduce the potential influence of researcher bias, questions were formulated to be as neutral and open-ended as possible (25). Accordingly, we acknowledge that the findings presented in this paper reflect interpretations that were inevitably influenced by the perspectives of both the participants and the research team.

Two independent coders (SB: researcher, female; and RdV; research assistant, male) analysed the transcripts and identified preliminary codes. The primary researcher (SB) has a background in clinical neuropsychology with a focus on supporting people with dementia to live at home for as long as possible, and the second coder (RdV) works as a junior researcher and neuropsychologist at an Alzheimer Center. To enhance reflexivity, the coders had regular meetings to compare and refine the codes, discuss interpretations and assumptions, and reach consensus on the broader coding categories and subcategories. In the instances where disagreement arose, the coders returned to the original data to seek additional context before reaching a final decision, and these discussions were documented as an audit trail. When consensus could not be reached, a third person (MM; lead researcher, male) was consulted. Thematic saturation was determined during analysis, indicated by the absence of new distinguishable themes or subthemes emerging from the data.

The data of the dyads and healthcare professionals were first analysed separately, but were also compared to explore differences and similarities between the participant groups. This allowed us to identify overlapping themes as well as group-specific perspectives. To explore contextual differences, participants were categorized based on their geographical location (rural vs. city settings), which was color-coded in the analysis. This supported our analysis of how geographical location may have shaped participants' experiences and whether certain themes were more prominent in specific geographical contexts. Finally, all categories were reviewed and grouped into overarching themes to capture the most relevant findings, resulting in the key themes reported in this paper.

## 3 Results

### 3.1 Participant characteristics

Thirty-five dyads participated in the study. About half of the people with dementia were female (18/35, 51%) and the average age was 77.7 years (SD = 7.0). Type of dementia varied: most people were diagnosed with Alzheimer's disease (11/35, 31%) or Dementia Not Otherwise Specified (11/35, 31%), and received the diagnosis 2 to 4 years before enrolling in this study. The diagnosis was most frequently established by a medical specialist, such as a geriatrician or neurologist (16/35, 46%), or a multidisciplinary team (10/35, 29%). The majority of the informal caregivers were female (21/35, 60%). The average age of the caregivers was 70.0 years (SD = 14.0). Most dyads were spouses (29/35, 83%), the rest were adult-children (6/35, 17%). An overview of the dyads' characteristics can be found in Table 1.

**Table 1:** Background characteristics of the persons with dementia and informal caregivers (*N* = 35).

Characteristic	Overall sample (*N* = 35)	
	Persons with dementia	Informal caregivers
Gender (female)	18 (51%)	21 (60%)
Age (in y)	77.7(7.0) [60–92]	70.0(14.0) [23–87]
Educational level	
Low education	5 (14%)	3 (9%)
Mid education	14 (40%)	12 (34%)
High education	16 (46%)	20 (57%)
Relationship to PwD	
Spouse		29 (83%)
Child		6 (17%)
Cohabiting		27 (77%)
Living area	
City		19 (54%)
Rural		16 (46%)
Type of dementia	
Alzheimer's disease	11 (31%)	
Dementia Not Otherwise Specified	11 (31%)	
Vascular dementia	5 (14%)	
Lewy body dementia	2 (6%)	
Frontotemporal dementia	2 (6%)	
Mild Cognitive Impairment	2 (6%)	
Other	1 (3%)	
Unknown at time of assessment	1 (3%)	
Time since diagnosis	
0–24 months	13 (37%)	
24–48 months	14 (40%)	
48+ months	7 (20%)	
No official diagnosis	1 (3%)	

Data depicted as *n* (%) or mean (standard deviation) and [range].

We included 47 healthcare professionals, where most worked as a dementia case manager (18/47, 38%). On average, healthcare professionals had 17 years of working experience in older adults care, which ranged from 1 to 41 years. The working area of the professionals covered rural as well as city areas throughout the Netherlands. Further characteristics can be found in Table 2.

**Table 2:** Background characteristics of the healthcare professionals (*N* = 47).

Characteristic	Overall sample (*N* = 47)
Female	41 (87.2%)
Educational level	
Mid education (10 years)	3 (6%)
High education (11 years or more)	44 (94%)
Function in older adults care	
Dementia casemanager	18 (38%)
District nurse	6 (13%)
Dementia daycare centre coordinator	6 (13%)
Dementia walk in centre coordinator	4 (8%)
Advisor in informal care	5 (11%)
General practitioner	3 (6%)
Other	5 (11%)
Work experience in older adults care (in y)	17 [1–42]
Work area	
City	21 (45%)
Rural	17 (36%)
Both	9 (19%)

Data depicted as *n* (%) or mean [range].

### 3.2 Themes

Data analysis resulted in 13 themes, which we could group into three main categories (see Table 3 for details):

**Table 3:** Overview of the themes divided over three main categories and their related sources.

Theme title	Source
Category: Needs in the early stage	
Theme 1: More information on dementia and its progression	D
Theme 2: Navigating resources	D
Theme 3: Someone who listens	D
Theme 4: Availability of a case manager	D
Category: Facilitators	
Theme 1: Collaborating with other organizations and maintaining short lines of communication	H
Theme 2: Community engagement and accessibility	D + H
Theme 3: Raising societal awareness through education	H
Theme 4: A personal approach by being patient and building trust	H
Category: Barriers	
Theme 1: Reluctance to seek help and caregiver role identity	D + H
Theme 2: Negative perceptions and stigma surrounding dementia	D + H
Theme 3: Limited awareness and accessibility of services	D + H
Theme 4: Bureaucratic hurdles and complex procedures	D + H
Theme 5: One-size-fits-all approaches do not work	H

Themes were based on data of people with dementia and their informal caregiver, indicated by D for dyad, or data of healthcare professionals, indicated by H.

- Early-stage needs of people with dementia and their informal caregivers: This category included themes related to the specific support deemed most helpful during this period, the best way that healthcare professionals could have provided assistance, and what particular type of support was valued most by the dyads.
- Facilitators for support: This category included all themes focusing on approaches currently implemented by healthcare professionals which were perceived as successful, with particular attention to possible distinctions between city and rural settings.
- Barriers for support: This category comprised themes that addressed the perceived challenges in finding help, and the type of support that the dyads did not find useful, as well as ineffective strategies used by healthcare professionals.

### 3.3 Early-stage needs of people with dementia and their informal caregivers

We asked participants to reflect on their experiences during the early stages of dementia. Most informal caregivers reported an increasing need for assistance as the symptoms progressed, yet approximately half the caregivers indicated that they did not seek professional support at the time, believing they could manage the situation on their own. Practical support, such as assistance with daily activities (ADL), and domestic help, was often the first type of assistance informal caregivers were willing to accept.

#### 3.3.1 Theme 1: more information on dementia and its progression

The majority of the caregivers reported a lack of knowledge about dementia during the early stages and emphasized the importance of receiving more information about dementia symptoms, the expected progression of the disease, and what to expect in the period following a dementia diagnosis.

“*[I needed] someone who understands what you are going through, and who is there for you. [I needed more] information about the disease, the possible course, how to communicate with my partner. Someone to guide us both, so that my partner also understands to some extent what it would mean for him and me.” (D_7/informal caregiver/62 years old/Female)*

“*After the diagnosis, I was immediately assigned a case manager, and that support has been invaluable to me – especially the help with arranging everything I wouldn't have thought of myself!” (D_35/informal caregiver/67 years old/Female)*

#### 3.3.2 Theme 2: navigating resources

More than half of the participants reported no difficulties in finding support. Many, especially those in rural areas, described a well-coordinated healthcare network, where healthcare professionals in the area collaborated to help dyads access support. This indicated a contextual difference regarding the coordination of support, where healthcare professionals in smaller communities often knew each other personally, facilitating collaboration and referrals, and thus making it easier for dyads to find their way to support. Additionally, some dyads reported having prior experience with dementia within their family or surroundings, which provided them with the knowledge and confidence to navigate the system independently.

Participants who did report challenges most frequently reported a lack of knowledge regarding available services, often attributed to limited information provision by healthcare professionals. Many informal caregivers felt that healthcare professionals could have played a more proactive role in facilitating case management and other services. General practitioners and neurologists, in particular, were perceived as playing a passive role in facilitating access to support, with some being unaware of existing resources such as newly founded walk-in centres.

“*I found it strange that neither my general practitioner nor my dementia case manager was aware of the existence of the Odensehuis [walk-in centre for persons in early-stage dementia].” (D_1/person with dementia/77/M)*

#### 3.3.3 Theme 3: someone who listens

Across the sample, dyads emphasized the need for emotional support and a sense of being heard. Participants stressed the importance for healthcare professionals to establish trust through active listening and providing accessible and readily understandable information about the disease. Participants highlighted the importance of peer support and joint activities to reduce feelings of isolation and strengthen social connections.

“*Occasionally, I just needed a break—a chance to step away from the home situation, knowing that someone else could take over the responsibility and care for my husband.” (D_8/informal caregiver/79/F)*

#### 3.3.4 Theme 4: availability of a case manager

Several participants described difficulties in establishing contact with dementia case managers or care consultants, while others reported frequent changes in the case managers assigned to them. Such disruptions were perceived as confusing, particularly for the person with dementia, and contributed to a sense of discontinuity in care.

### 3.4 Facilitators for support

#### 3.4.1 Theme 1: collaborating with other organizations and maintaining short lines of communication

Healthcare professionals emphasized the importance of establishing strong communication with those responsible for referrals, such as general practitioners. Some organizations facilitated this by organizing information sessions for the healthcare professionals involved, helping to raise awareness of available support. Collaboration with domestic care or home help services was also effective, as these professionals are often the first to interact with care recipients and can help identify dementia symptoms early on.

“*Inform GPs that I will take on a case without this being extra work for the GP.” (P_15/coordinator walk in centre/city)*

“*[…] maintaining short lines of communication is crucial, because then the general practice professional (“POH”) can call me immediately if something seems off [with the patient]. There is a strong sense of mutual involvement, and everyone has to set aside their organizational roles – we work together as one, for the people.” (P_22/informal care advisor/rural)*

Healthcare professionals also stressed the importance of actively expanding their professional network by attending community events such as information sessions, workshops and working groups in older adults care, to stay informed about local resources and ensure easier referral pathways between organizations.

#### 3.4.2 Theme 2: community engagement and accessibility

Healthcare professionals emphasized the importance of a visible presence within the community to facilitate access to support. Active participation in local initiatives, such as Alzheimer cafés or meetups, was recognized as an effective strategy for initiating early conversations with the dyad about care options. The physical accessibility of support service locations was also frequently emphasized, with professionals stressing that healthcare centres and walk-in facilities should be situated in familiar and accessible areas, rather than clinical settings. Dyads confirmed that the physical visibility and accessibility of services lowered their threshold for seeking support.

“*Working from community centres allows for earlier detection through activities, making it easier to identify emerging issues. The threshold for seeking support is lower, because you're already a familiar face [to them]. [...] Many people initially joined as volunteers at the community centre and later became clients or took on a dual role as both volunteer and client. Being visible as much as possible within the neighbourhood remains essential.” (P_43/social district support team/city)*

Continuous outreach was deemed crucial, as healthcare professionals noted that people typically seek support only when they feel ready. Flyers and websites appeared to be less effective; healthcare professionals rely more on word-of-mouth referrals to encourage people with dementia and their families to seek support.

“*And you have to continuously work towards raising awareness – it is not enough to do it once or twice a year. You really have to keep it on people's radar. In my experience, people eventually take notice but they often think information is not relevant to them at that moment. Then, a few months later, they become more receptive.” (P_42/case manager/both city and rural)*

#### 3.4.3 Theme 3: raising societal awareness through education

Healthcare professionals described actively working to raise awareness of dementia to make an impact by giving presentations to a variety of groups, including local religious communities, migrant populations, bus and supermarket staff, schoolchildren, and pharmacists. These efforts aim to enhance awareness not only among people directly affected by dementia, but more broadly across society with the goal of creating a more dementia-friendly community in which people can recognize dementia symptoms earlier and feel less stigmatized in seeking help. It is also crucial to inform older adults themselves about the various options of care available once a dementia diagnosis is made. By engaging diverse communities and spreading knowledge, healthcare professionals are working toward a greater understanding of dementia, encouraging earlier intervention and reducing stigma.

“*We try to raise as much awareness as possible to the fact that help [for someone dealing] with dementia exists. We give talks to a broad audience and give guest lectures at secondary schools. We also provide education to bus drivers and people in the neighbourhood, aiming to increase awareness in an accessible way for everyone in society. We contribute to creating dementia-friendly municipalities.” (P_42/case manager/both city and rural)*

#### 3.4.4 Theme 4: a personal approach by being patient and building trust

Healthcare professionals emphasized the importance of low-threshold access, where no official registration or referral is required to initiate contact. Providing a welcoming environment where people can freely express concerns, even before an official diagnosis, is key to facilitating early engagement. Engaging informally, such as over coffee or during a walk, was highlighted as an effective way to build trust. Professionals emphasized the need for having patience and an empathetic approach, prioritizing active listening over immediately offering solutions to create a safe and non-judgemental environment, encouraging people to seek help when they are ready.

“*Being accessible – [that people are able to] just drop by for a cup of coffee. Freedom is also important: the freedom to participate without having an official diagnosis or feeling obligated to take certain steps. The fact that people can come to the Odensehuis together also helps. Some couples deliberately visit as a pair simply because they enjoy spending a pleasant morning together.” (P_23/coordinator dementia walk in centre/rural)*

### 3.5 Barriers for support

#### 3.5.1 Theme 1: reluctance to seek help and caregiver role identity

Healthcare professionals indicated that a key barrier to early help-seeking was that informal caregivers must first come to terms with their role as caregivers before they can start seeking help. Dyads confirmed that they were often caught between wanting to manage the situation independently and recognizing that it was becoming unmanageable, as they felt that caregiving was part of their familial role. This barrier was particularly evident in rural areas, where caregivers in these highly interconnected communities were often reluctant to disclose their struggles, as they wished to maintain a capable image to the outside world out of fear of stigma. This reluctance typically resulted in postponing the diagnosis, which in turn delays the start of support services, leaving little time for the healthcare professional to build trust or properly address the needs and preferences of the dyads.

“*The partner does not see themselves as caregiver. Caregivers often feel they must handle [the situation] on their own. The boundaries [of caregiving] keep shifting, and they only realize there is a problem once they have already gone too far. By then, the situation has escalated.” (P_2/coordinator dementia daycare centre/city)*

Caregivers were often hesitant to seek help due to concerns that it may lead to immediate and significant changes in their lives, for instance that the person with dementia may no longer be able to live at home or be allowed to drive, or that seeking help might lead to the involvement of numerous external agencies.

#### 3.5.2 Theme 2: negative perceptions and stigma surrounding dementia

Beyond individual reluctance, broader societal stigma surrounding dementia presented a significant barrier. Some people with dementia were aware that they might have dementia but hesitated to disclose this due to concerns of how society would perceive and treat them following a dementia label. Dyads described how such a label had changed the way others related to them, which had made them more cautious about disclosing their situation. They often described a shift in social interactions following diagnosis, where professionals confirmed that people have the tendency to start talking about a person with dementia rather than addressing them directly, contributing to feelings of isolation and a loss of self-worth in the person with dementia.

“*When Piet and Jan go to the Odensehuis together, and Piet has dementia, people will ask Jan how Piet is doing. You are no longer included in the conversation, they stop seeing you as a person. But that is not true – you simply have dementia.” (P_12/coordinator dementia walk in centre/rural)*

As a result of the stigma, healthcare professionals noted lower attendance at events explicitly labeled with the terms “dementia” or “Alzheimer” as it can be discouraging. Some professionals responded by reframing their services using less stigmatizing language.

“*In [our region], for example, support is provided in the community centre where we use the term “struggling brain” instead of “dementia”.” (P_43/social district support team/city)*

#### 3.5.3 Theme 3: limited awareness and accessibility of services

Healthcare professionals in both city and rural areas reported significant challenges in reaching people in need of support. A key issue is the lack of awareness regarding available services among dyads themselves, despite the many services available. People with dementia and their caregivers often struggled to find the right support at the right time, partly due to inadequate and inaccessible information provision. Many older adults struggled to access information because it is primarily available online and requires digital skills, presenting a barrier for a generation that did not grow up with modern technology. The language used in the information materials was also often perceived as too complex or acts as a deterrent and should be more accessible and simpler to ensure that the information reaches those who need it most.

“*Highly educated older adults – or their well-educated children – are often able to find support. However, those with lower educational backgrounds find it much more complicated. I believe this is the group that should be prioritized, and information should be provided in much simpler language.” (P_21/coordinator dementia daycare centre/city)*

#### 3.5.4 Theme 4: bureaucratic hurdles and complex procedures

Administrative burdens and the complexity of applying for services, such as the *WMO* (“Social Support Act”) or *WLZ* (“Long-Term Care Act”), deter people with dementia or caregivers from seeking support. Long waiting lists for case management, especially in city areas, and frequent policy changes within healthcare legislation and organization created unnecessary complexity for both care providers and recipients. Dyads similarly reported encountering difficulties navigating the administrative requirements for accessing (formal) care, often making them hesitant to pursue support.

These challenges were consequently more pronounced in city areas, where high caseloads limited professionals' capacity for additional activities or personalized care, often resulting in superficial interactions. If a dementia case manager is not immediately assigned to the dyad, the only contact after diagnosis was typically a brief check-in from the general practitioner twice a year. In contrast, healthcare professionals across our sample expressed a preference for a multidisciplinary approach that would provide continuous support from the initial signs of concern (“niet-pluis”) through to end-of-life care. In rural areas, collaboration between various organizations was frequently described as better established, whereas professionals in the city consistently noted a fragmented system where different providers often operate in isolation, almost fostering competition between healthcare professionals rather than cooperation.

“*If you look at the Dementia Care Standard, support should already begin when there is a feeling that something is not right (“niet-pluis”). It is frustrating to witness the [personnel] shortages in the workplace, as we want to help everyone who needs it, but that it simply not possible due to overwhelming waiting lists.” (P_46/dementia case manager/city)*

#### 3.5.5 Theme 5: one-size-fits-all approaches do not work

Healthcare professionals, particularly in city areas, highlighted the limitations of standardized support programs, which often failed to meet the diverse needs of the dyads. The professionals also noted that if a dyad feels pressured, it tends to be counterproductive for accepting care or support. They stressed the need to be flexible and creative in order to engage people, for example by starting with informal activities like a walk, gradually building up trust, and introducing support in a way that aligns with the dyad's preference and level of readiness. The professionals also noted that the current approach of providing care is focused too much on what the person with dementia can no longer do, rather than exploring their remaining strengths and abilities, which may cause care recipients to present themselves as more capable than they actually are, further delaying access to support.

“*Providing something that is meant for everyone often means it works for no one – support should be tailored to individual needs and capabilities.” (P_32/informal care advisor/city)*

## 4 Discussion

This study explored the needs, facilitators, and barriers to early-stage dementia care of people with dementia, informal caregivers, and healthcare professionals in both city and rural settings. A central finding was that informal caregivers in the early stages of dementia frequently underestimated their need for professional support, despite facing increasing challenges in caring for their loved ones. This is consistent with previous findings, where caregivers retrospectively indicated they would have benefited from someone to talk to and from earlier recognition of their problems (19, 26). In earlier research, this phenomenon has been labeled as the early stage needs paradox (19): caregivers in the early phase struggle to acknowledge their need for care due to difficulties in accepting changes and fear of stigma, as well as a preference to resolve matters independently. This aligns with the notion that caregivers' reluctance is not only driven by a lack of information, but also by personal values around independence and role identity, because accepting help may feel like acknowledging a loss of control that many caregivers in the early phase are not yet ready to face. The current study adds to this perspective by showing that professionals were well aware of this paradox, and thus actively adapted their outreach strategies in response by building trust gradually and using informal contact moments. Furthermore, our findings suggest that framing support explicitly as caregiver support may be counterproductive in the early stages, as many people do not yet identify with that role. This may partly explain why practical assistance, such as domestic help or support with daily tasks, often appeared to be the first form of support caregivers were willing to accept and may therefore serve as a valuable entry point for early engagement that feels less intrusive than interventions explicitly labeled as dementia care.

In our study, dyads emphasized the importance of timely, clear information on dementia, particularly regarding symptom progression, available services, and navigating care. Healthcare professionals similarly noted that inadequate information provision and complicated procedures for accessing care hindered awareness of available services, especially among people with lower literacy or limited digital skills, which tend to be more prevalent in the current older population (27). Earlier research has found that limited knowledge of and the complexity of the healthcare system can delay help-seeking (26). The role of general practitioners in recognizing dementia symptoms early and facilitating timely referrals has also been identified as a key factor in improving early-stage care access, including in primary care research on dementia preparedness (15, 16). Beyond information, dyads expressed a strong need for emotional support and a sense of being heard. Case management was identified as a critical need across our sample, aligning with the latest report on the wishes and needs of people with dementia and their caregivers in the Netherlands (28).

Healthcare professionals in our study emphasized the importance of strong collaboration with other healthcare organizations and maintaining clear lines of communication, particularly with general practitioners and community-based services. In both rural and city areas, a visible presence in the community, through walk-in centres, local events, or informal networks, was described as crucial for lowering the threshold for contact. Educational efforts aimed at diverse community groups were seen as key for improving recognition and acceptance of dementia, consistent with research showing that public awareness campaigns are central to creating inclusive, dementia-friendly communities (29) and lowering the threshold for seeking an early diagnosis (30). Importantly, professionals stressed that community visibility alone is insufficient without a personal approach: low-threshold, trust-based contact, such as informal conversations over coffee or during a walk, was considered essential for engaging dyads who are not yet ready to accept formal care. This approach directly mirrors caregivers' preferences for informal, non-judgemental contact that does not immediately imply a transition to formal care, suggesting that aligning professional outreach strategies with the readiness and preferences of dyads may help bridge the gap between available services and actual uptake.

While professionals described several promising strategies for early engagement, our findings also revealed that systemic barriers risk undermining these very efforts. Bureaucratic procedures, frequent policy changes, and long waiting lists deter people from seeking support at precisely the moment professionals are trying to build trust and maintain continuity. Across both facilitators and barriers, a recurring contextual pattern emerged, where professionals and dyads in rural areas generally described better-coordinated, more personal care networks, whereas those in the city areas more frequently encountered fragmented systems, higher caseloads, and greater anonymity in care relationships. Dyads, especially in the city area, often struggled to navigate the healthcare system, where the absence of a familiar face or central point of contact led to delayed engagement (26). High caseloads in city settings further limited professionals' capacity for the personalized, flexible approach they identify as essential (31). This tension between what professionals know works and what the system allows them to do represents a structural mismatch that is consistent with broader European research, where fragmented services, high staff turnover, and poor coordination were among the most consistently reported organizational barriers to dementia care (32, 33). One-size-fits-all approaches similarly failed to meet the specific needs of dyads (34). Such systemic shortcomings actively undermine the continuity and trust-based aspects of care. Policy reform should therefore go beyond procedural simplification and address the structural conditions that enable professionals to build and maintain meaningful relationships with dyads over time, such as manageable caseloads, reduced administrative burden, and stronger incentives for interdisciplinary collaboration. In the Dutch context, existing frameworks such as the Dementia Care Standard and the Social Support Act (WMO) may serve as valuable entry points for such reform, especially because professionals stressed that such efforts require time, resources, and continuity: conditions not always met within the current care infrastructure (31).

Despite a growing emphasis on early intervention, many dyads in our sample still postponed seeking support, not solely due to a lack of knowledge, but also due to personal values concerning role identity, independence, fear, and stigma. Previous research has identified emotional denial as an important barrier for seeking help in early-stage dementia (35). Our findings suggest that stigma may be more pronounced in rural settings, where the interconnected nature of these communities meant that caregivers feared their struggles would become known, which made formal help-seeking feel more exposing. This is consistent with previous research on rural dementia care, which suggests that such social structures can both support and hinder help-seeking behavior (36). Caregivers similarly expressed hesitation in seeking help, fearing it might result in immediate and substantial changes to their lives, yet earlier research has shown that seeking help at a later stage is associated with reduced involvement of the person with dementia in decision-making concerning daily support (3), suggesting that the very act of postponing care may undermine the autonomy dyads are trying to protect. Healthcare professionals highlighted the impact of stigma and societal perceptions, echoing what caregivers and people with dementia described as changes in social interactions and a fear of being labeled following a diagnosis (37). Consequently, a vicious cycle appears to emerge, where limited awareness of services and the stigma surrounding dementia contribute to fear of seeking help or even seeking out a diagnosis.

In sum, the findings of this study show that professionals and dyads often approached the early phase differently. Where professionals described it as a window for proactive engagement, dyads more frequently experienced it as a period of adjustment in which professional support was deemed unnecessary or premature. This divergence was further shaped by geographical context, with rural settings fostering more personal, proactive outreach but it was also made harder to seek help without others noticing, while city settings offered more anonymity but less continuity and navigational support. These insights suggest that improving early-stage care acceptance requires attention not only to what is offered, but also to how, when, and where it is offered in relation to the readiness of the dyad.

## 5 Strengths and limitations

A strength of this study is the inclusion of multiple perspectives from informal caregivers, people with dementia, and healthcare professionals. This approach provides a comprehensive understanding of the needs and challenges in early-stage dementia from both care recipients and providers. The study looked at the everyday strategies professionals use to engage people with dementia and informal caregivers, as well as systemic barriers, offering practical insights for improving outreach and accessibility of care. Furthermore, the inclusion of participants from both city and rural areas strengthens the transferability of our findings. Comparing city and rural areas is a novel contribution to the literature, which showed how geographical context influences access to care, support-seeking behavior, and availability of services. By focusing explicitly on the early stages of dementia, this study addressed a phase that is often underrepresented in dementia research, despite its significance for timely intervention and quality of life of the target group.

Some limitations should be noted. There may have been a potential recall bias (38), as the dyads who agreed to participate might have been more proactive and had more engagement with healthcare professionals. This could have possibly led to an underrepresentation of those who were more isolated or harder to reach, potentially resulting in differences in reported needs or barriers. Additionally, reflecting retrospectively on the early stage may have been influenced by the current experiences and hindsight of the dyads, shaping how they remember their earlier experiences (19, 38). Furthermore, clinical characteristics were limited to general descriptors. The lack of objective clinical assessments limits our ability to link specific cognitive profiles to the experienced needs, facilitators and barriers. Lastly, we did not return the interview transcripts to the healthcare professionals for comments or feedback, in order to not further burden them during the COVID-19 pandemic, which means our interpretations of their answers may differ from their intended meanings.

## 6 Future directions for research and practice

The findings suggest that effective early-stage support may require a reframing of what care means. Rather than positioning support as a way to deal with decline, presenting it as a way to maintain agency, continue meaningful activities, and maintaining quality of life may lower the threshold for acceptance, particularly for caregivers who feel responsible to handle the situation on their own. To facilitate this shift, it may be beneficial to adopt a more positive perspective of dementia, recognizing the abilities of people with dementia in addition to their losses. Community-based, low-threshold initiatives such as Meeting Centres (39–41), Odensehuizen, the Social Approach to Dementia (42), and DemenTalent (43) exemplify how this reframing can be put into practice, by focusing on strengths and social participation rather than loss.

In city areas, care structures tend to be fragmented, resulting in limited continuity and longer waiting times for case management. In contrast, dyads in rural areas frequently described a proactive and well-connected network of healthcare professionals that facilitated smoother access to services. These insights highlight the importance of local context in designing interventions and implementing these in dementia care pathways. Future research should explore whether interventions can be better aligned with their geographical infrastructure. It would also be beneficial to investigate how collaboration across disciplines and organizations could be enhanced to promote continuity and accessibility in early-stage dementia care. Additionally, outreach strategies tailored to underrepresented groups should be examined, including individuals with low literacy or limited digital access, migrants, socially isolated caregivers, and people with dementia lacking a support network. Particular attention should be paid to the role of language and labeling, as the stigma associated with a dementia diagnosis may influence whether people engage with available support at all.

## 7 Conclusion

This study examined the needs, facilitators, and barriers to early-stage dementia care from the perspectives of people with dementia, informal caregivers, and healthcare professionals. The integration of these three perspectives revealed that delays in seeking support are driven not only by limited awareness of services, but also by emotional readiness, stigma and fear surrounding dementia, and personal values around role identity and independence: factors that interact in complex ways and might not be addressed through a single intervention or approach. Practical access is further obstructed by bureaucratic procedures and fragmented care systems, especially in city settings, while regional variations in healthcare professional networks suggest that local context can significantly influence access to care. Together, these insights call for approaches that acknowledge this complexity, such that person-centred, context-sensitive support reframes early-stage care not as a response to decline, but as a way to maintain agency, social participation, and quality of life. Importantly, our findings suggest that trust-based, relational approaches, characterized by patience, personal contact, and low-threshold access, are central to facilitating early-stage care acceptance, and that these can only be effective when systemic conditions, such as continuity of care and manageable caseloads, allow professionals to work in this way.

## Data Availability

The datasets presented in this article are not readily available because the datasets generated and/or analysed during the current study will not be shared due to privacy reasons. Requests to access the datasets should be directed to s.c.e.balvert@vu.nl.
