# Supplementary material for: Facilitators and barriers to early-stage dementia care: a qualitative study on the perspectives of people with dementia, informal caregivers, and healthcare professionals
Source: Front Public Health. 2026 Jul 6;14:1867784. doi: 10.3389/fpubh.2026.1867784 (PMC13381491; doi:10.3389/fpubh.2026.1867784)
Supplement: Supplementary file 1 [file Table_1.docx]

‘Eerder Erbij’: how can we reach people with dementia and their caregivers in an earlier phase?

1. **In which region do you work?**

City: ………………………………………….……………………………………………………………………………………..

Province: ………………………………………….……………………………………………………………………………………..

1. **What is your highest level of education?**

- MBO
- HBO
- WO
- Other: …………………………………………………………………………………………………………….

1. **Did this level of education fall within the sector elderly care?**

- Yes, namely:
- No

1. **What is your role within elderly care?**
2. **How long have you been working in this position?**
3. **How long have you been working in elderly care overall?**

The following questions are about how your organisation comes into contact with new clients. How are new clients referred or registered at your organisation? Think, for example, of collaboration with dementia case managers, general practitioners, domestic care providers, district nurses, or other (welfare) organisations.

1. **How do people with dementia come into contact with your organisation?**
2. **Which other organisations do you collaborate with in this regard?**

The following questions are about what your organisation does to reach people in an earlier phase of the dementia. Think, for example, or preventive home visits, training for recognising early warning signs (in Dutch ‘niet-pluis gevoel’), collaborations within the neighbourhood or walk-in centres, or inviting people with memory problems to volunteer within the organisation. What does your organisation do beyond the “standard routes”?

1. **What does your organisation do to reach people with dementia (at an earlier stage)?**
2. **Which strategies have proven successful in this respect? What makes it easier to reach people?**
3. **Which strategies have not been successful? What makes it harder to reach people?**

The following questions concern the needs of people with dementia and their caregivers when they first come into contact with your organisation. This refers to the very early phase in which they begin using services such as domestic care or day care.

1. **When people with dementia and their caregivers first begin using professional assistance, what is particularly important during this initial phase?**
2. **Based on your experience in elderly care, when do you think is the right time for professional assistance? Or when is assistance too late?**

The following questions are about any courses or support currently offered within your organisation. For example, think of peer support, educational programmes or respite care. What type of support does your organisation already provide?

1. **What types of support or services does your organisation provide for people with dementia?**
2. **What types of support or services does your organisation provide for caregivers of people with dementia?**
3. **Does your organisation also offer courses or programmes specifically designed for both people with dementia and caregivers together?**Think for example of group meetings for both.

We are currently in the process of developing support for people with dementia and their caregivers. The following questions concern the general needs of your (new) clients. For example, think of a consistent point of contact within the care system, accessibility of services, a focus on positivity, continuity of care, strengthening the social network or alignment with personal preferences.

1. **What information and support do your clients need? What should be the focal points for a new kind of support approach?**
2. **What kind of information and support would you yourself like to offer to (new) clients? What do you think is best for people in this (earlier) phase of dementia?**
3. **When, in your opinion, will people with dementia and their caregivers have no need for information and support?**

Think for example about diagnosis may not yet be accepted, the caregiver may be overburdened or the person with dementia and their caregiver may be experiencing uncertainty or doubt.

1. **Is there anything else you would like to share?**
2. **Do you have any questions? If necessary, we can contact you.**

**Do you have any final comments or suggestions?**
