# Supplementary material for: Facilitators and barriers to early-stage dementia care: a qualitative study on the perspectives of people with dementia, informal caregivers, and healthcare professionals
Source: Front Public Health. 2026 Jul 6;14:1867784. doi: 10.3389/fpubh.2026.1867784 (PMC13381491; doi:10.3389/fpubh.2026.1867784)
Supplement: Supplementary file 2 [file Table_2.docx]

‘Eerder Erbij’: how can we reach people with dementia and their caregivers in an earlier phase?

We would like to ask you some general questions. You can answer the questions together.

The following questions concern the person with dementia.

**What is your age?**

………… years old

**What is your gender?**

- Female
- Male

**What is your highest level of education?**

- Less than sixth grade primary school (incomplete)
- Completed primary school
- More than primary school but no secondary or vocational education
- Trade school
- MULO/MMS/MAVO/secondary vocational education
- HBS/gymnasium/atheneum
- University/higher education

**Where do you live?**

City: …………………………….……………………………………………………………………………………………………..

Province: ………………………………………….…………………………………………………………………………..……………

**Have you been diagnosed with dementia?**

- No
- Yes

**If yes, what type of dementia were you diagnosed with?**

……………………………………………………………………………………………………………………………………………………………

**When was the diagnosis made?**

Year: …………………………….……………………………………………………………………………………………………..

**Who made the diagnosis?**

- General practitioner
  - Geriatrician
  - Neurologist
  - Memory clinic
  - Specialist in geriatric medicine
  - Other: …………………………………………………………………………………………………………………….

The following questions concern the informal caregiver.

**What is your age?**

………… years old

**What is your gender?**

- Female
- Male

**What is your highest level of education?**

- Less than sixth grade primary school (incomplete)
- Completed primary school
- More than primary school but no secondary or vocational education
- Trade school
- MULO/MMS/MAVO/secondary vocational education
- HBS/gymnasium/atheneum
- University/higher education

**What is your relationship with the person with dementia?**

- Spouse/partner
- Child
- Sibling
- Friend
- Other: ...................................................................

**How many other caregivers are involved in caring for the person with dementia?**

- 0
- 1
- 2
- 3 or more

**Besides caregiving, do you have other time-consuming responsibilities (e.g., work, other family responsibilities, volunteering)?**

- No
- Yes: …………………………………………………………………………………………………

**Do you live in the same house as the person with dementia?**

- Yes
- No

**If not, how often do you see or speak to the person with dementia (including phone contact)?**

……….. times a week

**If not, where do you live?**

- In the same region as the person with dementia
- Somewhere else: ……………………………………….………………………………………………………………………………

The following questions are for the person with dementia and caregiver together.

The following questions are about the period when the first problems began. This can be a difficult and uncertain time. We would like to know what support you would have found helpful during that phase.

1. **When did you first notice that more support was needed?**
2. **Would you have wanted more support at that time? Why or why not?**
3. **What kind of help would have been useful for you then?**

The next questions are about the period after the diagnosis. What kind of support did you need then, and what did you receive?

1. **What could healthcare professionals have done for you at that time?**
2. **What kind of support did you need then?** [multiple answers are possible]
   - Information
   - Someone to talk to
   - Social interaction
   - Peer support
   - Practical help
   - Time for yourself
   - Help choosing appropriate care
   - Other: ……………………………………………………………………………………………..
3. **What support did you actually receive?**
4. **Were you satisfied with that support? What did you appreciate, and what did you not appreciate?**
5. **How did you find this support?** [multiple answers possible]

- On my own (e.g., through local magazine or the internet)
  - Through a healthcare provider (e.g., general practitioner or case manager)
  - Through friends or family
  - Other: ………………………………………………………………………………………………………..

1. **Was it difficult to find this support?**

The following questions are about what kind of support you are currently using and what kind of support you find important.

1. **What support are you currently using?** [multiple answers possible]
   - Dementia case manager
   - Discussion groups/peer support
   - Individual guidance
   - Home care
   - Household help
   - Walk-in centre/Dementia support centre
   - Technology-based support
   - Day care centre
   - Meeting centre
   - Information about dementia (e.g., courses, websites, books)
   - Meal services
   - Respite care/short stay
   - Informal care (family, friends, acquaintances)
   - Other: …………………………………………………………………………………………………………………………
2. **What type of support is most important for you now?**

We are developing a support programme for people with dementia and their informal caregivers to attend together, especially when problems in daily life first emerge.

1. **Have you ever participated in such a course?**
   - **No**

**What was a reason for you to not participate in such a course?**

- - **Yes, the course:** ……………………………………………………………………………………………………………

**What was a reason for you to participate in such a course?**

**What did you learn from it?**

1. **When you think back to the period shortly after diagnosis, what topics would you have wanted more information about?** [multiple answers possible]

- Is dementia hereditary and what are the causes of dementia?
- Which types of dementia exist?
- Information about treatment plan: next steps after diagnosis
- What kind of support is available for people with dementia and caregivers?
- How to deal with emotions and tension
- Uncertainty, doubt and stigma
- Accepting and coping with the diagnosis
- Dealing with behavioural and cognitive changes
- Practical daily life support
- Coping with changes in the relationship
- Communicating with each other
- Coping with stress and fatigue
- How can I continue to take good care of myself?
- Staying connected with friends and family
- How can I approach dementia in a more positive way?
- Practical issues (e.g., driving and home modifications)
- Legal matters (e.g., advance directives, power of attorney, will)
- Financial matters (e.g., application for the Long-Term Care Act)
- End of life decisions (e.g., resuscitation and euthanasia)
- Other: ..............................................................................................................

1. **Are there any important topics that we have not covered?**
2. **Is there anything else you would like to share?**
3. **Do you have any questions? If necessary, we can contact you.**
4. **Would you be interested in participating in a group interview to discuss these topics further?**
   - No
   - Yes, you can reach me at telephone number: ………………………………………

**Do you have any final comments or suggestions?**
